# Cost-effectiveness of a school-based health promotion program in Canada: A life-course modeling approach

**DOI:** 10.1371/journal.pone.0177848

**Published:** 2017-05-18

**Authors:** John Paul Ekwaru, Arto Ohinmaa, Bach Xuan Tran, Solmaz Setayeshgar, Jeffrey A. Johnson, Paul J. Veugelers

**Affiliations:** 1 School of Public Health, University of Alberta, Edmonton, Alberta, Canada; 2 Institute for Preventive Medicine and Public Health, Hanoi Medical University, Hanoi, Vietnam; McMaster University, CANADA

## Abstract

**Background:**

The Alberta Project Promoting active Living and healthy Eating in Schools (APPLE Schools) has been recognized as a “best practice” in preventing childhood obesity. To inform decision making on the economic implications of APPLE Schools and to justify investment, we evaluated the project’s cost-effectiveness following a life-course approach.

**Methods:**

We developed a state transition model for the lifetime progression of body weight status comparing elementary school students attending APPLE Schools and control schools. This model quantified the lifetime impact of APPLE Schools in terms of prevention of excess body weight, chronic disease and improved quality-adjusted life years (QALY), from a school system’s cost perspective. Both costs and health outcomes were discounted to their present value using 3% discount rate.

**Results:**

The incremental cost-effectiveness ratio(ICER) of APPLE schools was CA$33,421 per QALY gained, and CA$1,555, CA$1,709 and CA$14,218 per prevented person years of excess weight, obesity and chronic disease, respectively. These estimates show that APPLE Schools is cost effective at a threshold of ICER < CA$50,000.

In probabilistic sensitivity analysis, APPLE Schools was cost effective more than 64% of the time per QALY gained, when using a threshold of ICER<CA$50,000, and more than 93% of the time when using a threshold of ICER<CA$100,000.

**Conclusion:**

School-based health promotion, such as APPLE Schools is a cost-effective intervention for obesity prevention and reduction of chronic disease risk over the lifetime. Expanding the coverage and allocating resources towards school-based programs like the APPLE Schools program, is likely to reduce the public health burden of obesity and chronic diseases.

## Introduction

Childhood obesity has become a public health priority in Canada and other developed countries [1]. Childhood obesity negatively affects children’s development and quality of life [2], and increases childhood health care costs[3]. It also increases the risk of obesity, chronic diseases and health care costs in adulthood [4]. Societal cost[5] estimates for obesity in Canada range from 1.27 to 11.08 billion Canadian dollars annually[6]. An estimated 2.2% to 12.0% of Canada’s total health care expenditures are consequences of excess body weight [6].

Inadequate physical activity and poor nutrition are underlying causes of obesity [7]. Promotion of active living and healthy eating is considered more effective when targeting children, and schools have been suggested as an ideal setting for health promotion as this is the place to reach nearly all children [8–12]. In 2005, we reported on a successful school-based project that achieved a reduction in the prevalence of excess bodyweight among children in Nova Scotia, Canada [13, 14]. The successful project was recognized as a “best practice” in Canada and inspired the development of the Alberta Project Promoting active Living and healthy Eating (APPLE) Schools in another Canadian jurisdiction [8]. APPLE Schools project was also successful in improving diets and physical activity levels, and in reducing obesity rates [8, 15]. Despite the recognition as ‘best practice’, public health decision makers have been hesitant to invest in large scale applications. They desire evidence on the long-term benefits such as prevention of obesity throughout adulthood and reduction in chronic diseases. Public health decision makers are particularly interested in program costs and potential cost savings resulting from avoided health care costs.

In a recent systematic review of economic evaluations of health promotion programs for children and adolescents, Korber[16] identified twelve economic evaluations of school-based programs in developed countries. However, none of these studies had been conducted in Canada, yet ‘local’ evidence is key to public health decision makers because countries differ with respect to both educational and health care sytems [6]. To better inform public health decision makers, we conducted an incremental cost-effectiveness analysis of the APPLE school program comparing the lifetime health outcomes to the program costs compared to an alternative where no program exists.

## Methods

We developed a Markov model to estimate the incremental cost-effectiveness ratio (ICER) for two strategies: APPLE Schools (the intervention program) compared to general schools (No intervention). The ICER was calculated by dividing the incremental cost of the school program by the incremental outcomes throughout the lifetime up to 80 years among male and 84 years among female (i.e ICER = [program cost-0]/[outcome with intervention program–outcome with No intervention program]). The outcomes considered were excess body weight (i.e., overweight and obesity), obesity, chronic disease and quality-adjusted life years (QALY). The QALY outcome combines the changes in both mortality (life years) and the health related quality of life due to morbidity. The $50,000 per QALY willingness-to-pay threshold has been an arbitrary decision rule since 1992 and is still used by many to determine if an intervention is cost-effective[17]. Based on the study of Shiroiwa et al. (2010)[18], $100,000 per QALY was chosen as a threshold for weak evidence of cost-effectiveness.

### The intervention program

Details of the intervention program are presented elsewhere [8, 19, 20]. In briefly, the APPLE Schools intervention involved having a full-time School Health Facilitator in each school for two years to implement healthy eating and active living policies, practices and strategies while engaging students, parents, school staff and other stakeholders. With the objective of changing the “school culture” to make the healthy choice the easy choice, the SHF facilitator contributed to the schools' health curriculum, both during instructional and non-instructional school time, developed cross curriculum links, and facilitated professional development days for teachers and school staff. Examples of activities include nutrition programs such as cooking clubs and healthy breakfast, lunch and snack programs, after school physical activity programs, walk-to-school days, community gardens, weekend events, school newsletters and daily health announcements. While the intervention was school wide, the evaluation focused on grade five students (about 10 years of age) [8]. The same surveys and measurements, including height and weight, were conducted in a sample of 148 randomly selected schools in Alberta allowing for a comparison of changes in APPLE Schools with average changes in control schools, as described in detail elsewhere [8].

### Conceptual framework and modeling approach

**Fig 1:**
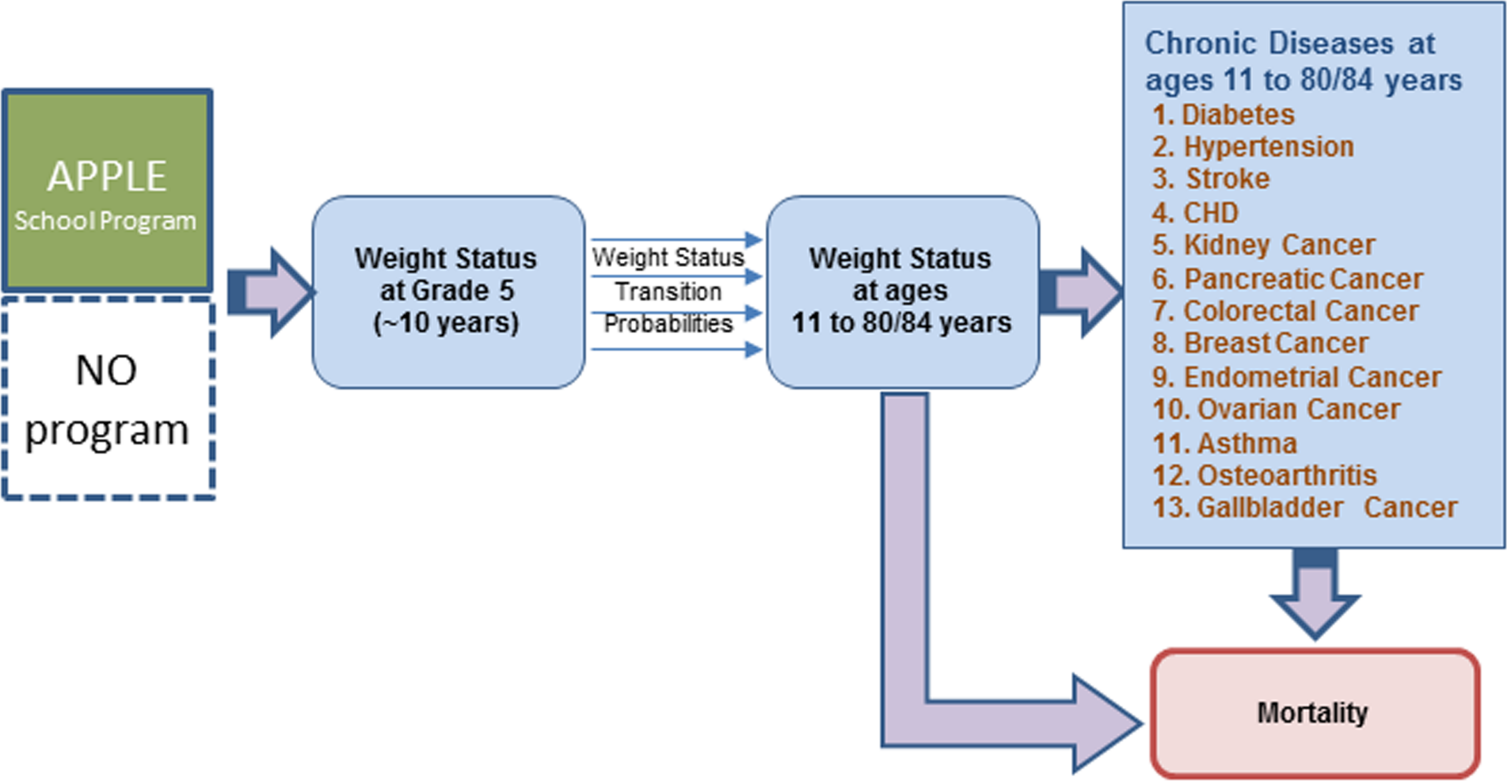
Model conceptual framework. Note: 80 and 84 are the life-expectancies at birth in British Columbia (the highest of all Canadian provinces) for men and women, respectively [26].

The conceptual framework for evaluating lifetime benefits of students attending APPLE Schools was based on evidence that body weight status at young age determines body weight in adulthood which in turn determines the risk of chronic conditions and health related quality of life [21–25]. Therefore, the effect of APPLE Schools, which was expressed in changes in weight status distribution of grade 5 students attending APPLE Schools relative to those attending control schools, was expected to affect lifetime weight status trajectories and risk of chronic diseases (Fig 1).

We assumed that the ‘school culture’ and lifestyle developed in the two intervention years would continue for another eight years. The model is therefore, based on 10 cohorts of students who pass through grade 5 over a 10-year period.

The model included 43 states based on 3 weight status categories (normal weight, overweight, and obesity), 13 chronic diseases with established links to weight status (i.e Diabetes, Hypertension, Asthma, Osteoarthritis, Stroke, Coronary Heart Disease (CHD), Kidney cancer, Pancreatic cancer, Colorectal cancer, Breast cancer, Endometrial cancer, Ovarian cancer, and Gallbladder cancer) [27], no-chronic disease state and the dead state.

Because school based programs in Canada are mainly funded by the education system, the modeling was carried out from the school system’s cost perspective and was implemented in TreeAge Pro 2016 software [28].

### Model inputs

#### Weight status transition probabilities

We obtained estimates of these probabilities from analysis of longitudinal data from two Canadian cohorts: 1) the National Population Health Survey(NPHS) that follows participants 12 years of age or older, and 2) the National Longitudinal Survey of Children and Youth (NLSCY) that follows children under 12 years of age. In both cohorts, data were collected from participants every 2 years. A multinomial logistic regression model (which allows for more than two levels of the outcome variable) was fitted to data from the two cohorts. The independent variables/covariates in this model were; sex, weight status and age at a given time point, and the outcome was weight status in the next assessment (2 years later). Parameter estimates of the fitted multinomial logistic regression model (S1 Table) were then used in the Markov model to obtain sex and age specific weight status transition probabilities.

#### Conditional probability of dying given weight status and chronic disease status

We estimated these probabilities based on general Canadian population mortality hazards by sex and age extracted from the Canadian Life table [26] and the effects of weight status (S2 Table) [29] and chronic diseases (S3 Table) [30–35] on all-cause mortality. Because the effects of weights status and chronic diseases on all-cause mortality were not estimated relative to the general population, the weight status distribution (S4 Table)[36] and prevalence of the various chronic diseases(S5 Table)[37–39], were also considered in the estimation.

At each cycle (age), the sex, age and weight status specific mortality hazard was estimated using the equation:

$$h_{w,s,a}=H_{s,a}\times \frac{{RR}_{w,s,a}}{\sum P_{w,s,a}{RR}_{w,s,a}}$$

Where

*h*_*w,s,a*_ – Conditional probability of dying in 1 year given weight status(w), sex(s) and age(a)
*RR*_*w,s,a*_—Relative risk of dying for weight status w, given sex and age ((Normal weight being the reference status)
*P*_*w,s,a*_—Proportion of individuals who are in weight status w, given sex and age.
*H*_*s,a*_ – Mortality hazard given sex and age, obtained from the Canadian life table

This estimation approach was used for all relative risks/rate ratios for which the reference category was not the general population, including in the estimation of the conditional probability of dying given chronic disease status (using S3 and S5 Tables) and conditional probability of developing a chronic disease given weight status(using S4, S6 and S7 Tables).

For each given combination of weight status and chronic disease state, we used the higher of the two estimated probabilities of dying, because we did not have estimates of the interaction effects between weight status and chronic diseases.

#### Probabilities of developing chronic diseases

We estimated these probabilities from published data on incidence rates of chronic diseases (S6 Table) [31, 40–45], the effects of weight status on incidence of these chronic diseases from a meta-analysis by Guh et al (S7 Table) [27] and weight status distribution (S4 Table)[36]. We considered only chronic diseases for which the meta-analysis found a significant effect including Diabetes, Hypertension, Stroke, Coronary artery disease, Asthma, Osteoarthritis and 7 cancers (Kidney, Pancreatic, Colorectal, Breast, Endometrial, Ovarian and Gallbladder) (S7 Table) [27].

#### Effect of APPLE schools on weight status of grade 5 students

We estimated the effect of the APPLE schools from analysis of weight status data obtained from grade 5 students attending APPLE and Non-APPLE Schools in the years 2008 and 2010. Based on a multinomial logistic regression model, APPLE Schools were estimated to reduce the odds of obesity over normal weight by 0.723 times per year (OR = 0.723, 95%CI: 0.553–0.946) in the two years of program implementation.

**Fig 2:**
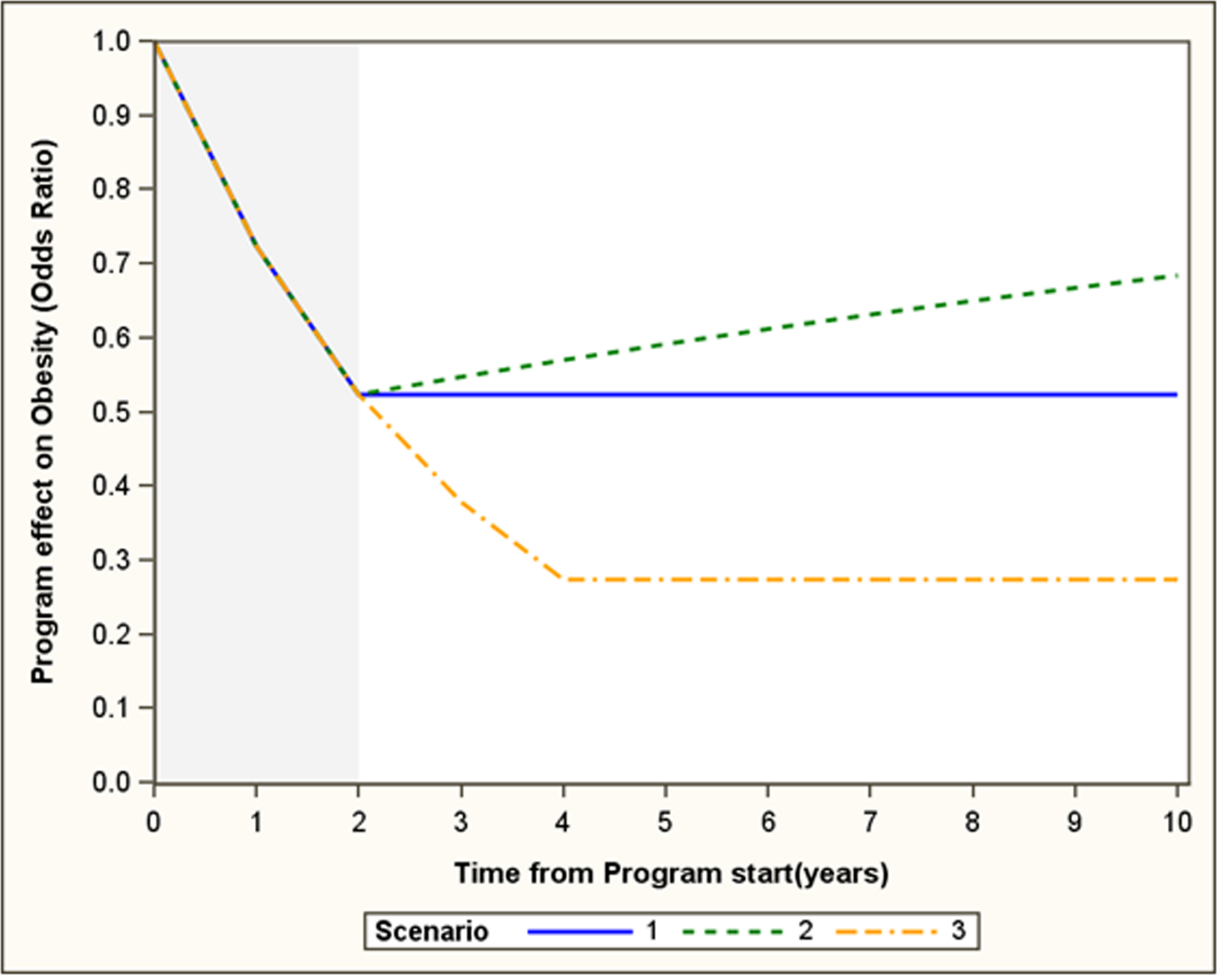
APPLE schools effects sustainability scenarios. Scenarios: 1-The annual decline in obesity is maintained for the 2 intervention years then remains at the level reached in 2 years for another 8 years 2- After the two intervention years, the effect starts waning by 5% per year 3- The annual decline in obesity is maintained for 4 years then remains at the level reached in 4 years for another 6 years.

Because the effect of the program was expected to continue for another 8 years, we considered three scenarios for the sustainability of the program effect after the two intervention years (Fig 2):

1. The annual decline in obesity is maintained for the 2 intervention years then remains at the level reached in 2 years for another 8 years(base scenario)
2. After the two intervention years, the effect wanes by 5% per year
3. The annual decline in obesity is maintained for 4 years then remains at the level reached in 4 years for another 6 years

#### Program costs

Using program accounts, we estimated the cost of APPLE Schools in the two year period (2008–2010) to be CA$284 per student per year in 2008 Canadian dollars (S9 Table). This estimate included all salaries of School Health Facilitators and managers, costs of professional development, programming, travel, and research costs.

The ICERs are reported in 2008 Canadian dollars. However for comparison with other published estimates, a conversation to 2011 US dollars was also carried out using estimates of National Consumer Price Indices (CPI) and Purchasing Power Parities (PPP) published by Organisation for Economic Co-operation and Development (OECD) [46, 47].

### Outcomes

We estimated the four outcome measures that were included in the model as person years of excess body weight (i.e., overweight and obesity), person years of obesity, person years with chronic diseases and QALY. For every year lived with excess weight, obesity or chronic disease we assigned a decrement in health utility scores producing QALYs lived with the condition. The estimated decrements in health utility score were obtained from previously published estimates from Schultz et al [48] and Jia et al [49] (S8 Table). Though the decrements estimated by Jia et al were based on participants who were 18 years of age or older, we applied the same decrements for children under 18 years as well. For each given combination of weight status and chronic disease Markov state, we used the higher of the two QALY decrement estimates (weight status or chronic disease).

### Discounting

All future costs (10 years) and health outcomes (up to 84 years) were discounted to their present values using annual discount rate of 3%[50].

### Sensitivity analyses

We conducted a number of sensitivity analyses to examine the robustness of the Markov model. In two-way sensitivity analysis, we varied the program effect scenario and discounting rate (0% and 5%).

We also performed probabilistic sensitivity analysis (PSA) to incorporate uncertainties in all model parameters simultaneously [51, 52]. In PSA, Relative risks and Odds ratios were assumed to follow log-normal distributions and parameters of the multinomial logistic regression model for weight status transitions were assumed to follow a normal distribution. Program costs were assumed to follow a gamma distribution and to have a coefficient of variation of 10%. In PSA, each model parameter was assigned a value drawn randomly from its distribution. We carried out 50,000 simulations of the model for each combination of scenario and outcome.

### Ethical consideration

This study has been reviewed and approved by the Health Research Ethics Board of the University of Alberta, Edmonton, Alberta, Canada (Protocol numbers: Pro0003799 and Pro00003800). Parents of the students who participated in the study had provided written informed consent and the students assented to the surveys and assessments of weight and height.

## Results

The estimated ICERs of APPLE Schools are presented in Table 1. In scenario 1 and assuming a discount rate of 3%, the model estimated that for every 10 children, 1.26 person years of excess weight (including 1.15 person years of obesity) and 0.14 person years of chronic disease would be prevented in their lifetime. This would correspond to a gain of 0.06 QALYs. In this scenario the ICER was estimated to be CA$1,555 per person year of excess weight prevented, CA$1,709 per person year of obesity prevented and CA$33,421 per QALY gained. Scenario 2 that assumed 5% annual reduction in the achieved effect after two years, the outcomes were slightly smaller leading to ICER of CA$40,396 per QALY gained. Contrary, scenario 3 that assumed 4 years of obesity decline the outcomes were higher resulting in an ICER of CA$23,216 per QALY gained.

**Table 1:** Cost-effectiveness of APPLE schools.

		YEARS WITH EXCESS WEIGHT		YEARS WITH OBESITY		YEARS WITH CHRONIC DISEASE		QALYs	
*SCENARIO*	Incremental Cost*	Incremental Effect*	*ICER*	Incremental Effect*	*ICER*	Incremental Effect*	*ICER*	Incremental Effect*	*ICER*
**Discount = 3%—Base results**									
1	1963.2	-1.26	**1,555**	-1.15	**1,709**	-0.14	**14,218**	0.06	**33,421**
2	1963.2	-1.04	**1,880**	-0.95	**2,065**	-0.11	**17,185**	0.05	**40,396**
3	1963.2	-1.82	**1,081**	-1.65	**1,187**	-0.20	**9,878**	0.08	**23,216**
**Discount = 0%**									
1	1988.0	-1.52	**1,312**	-1.36	**1,464**	-0.26	**7,599**	0.07	**28,530**
2	1988.0	-1.25	**1,586**	-1.12	**1,770**	-0.22	**9,185**	0.06	**34,485**
3	1988.0	-2.18	**911**	-1.95	**1,017**	-0.38	**5,279**	0.10	**19,819**
**Discount = 1%**									
1	1979.6	-1.42	**1,398**	-1.28	**1,552**	-0.21	**9,534**	0.07	**30,102**
2	1979.6	-1.17	**1,689**	-1.06	**1,876**	-0.17	**11,524**	0.05	**36,386**
3	1979.6	-2.04	**971**	-1.84	**1,078**	-0.30	**6,624**	0.09	**20,911**
**Discount = 2%**									
1	1971.3	-1.33	**1,479**	-1.21	**1,633**	-0.17	**11,742**	0.06	**31,761**
2	1971.3	-1.10	**1,787**	-1.00	**1,974**	-0.14	**14,192**	0.05	**38,390**
3	1971.3	-1.92	**1,027**	-1.74	**1,135**	-0.24	**8,158**	0.09	**22,063**
**Discount = 4%**									
1	1955.2	-1.20	**1,629**	-1.10	**1,779**	-0.12	**16,954**	0.06	**35,037**
2	1955.2	-0.99	**1,968**	-0.91	**2,150**	-0.10	**20,492**	0.05	**42,350**
3	1955.2	-1.73	**1,131**	-1.58	**1,236**	-0.17	**11,779**	0.08	**24,339**
**Discount = 5%**									
1	1947.4	-1.15	**1,698**	-1.06	**1,844**	-0.10	**19,938**	0.05	**36,590**
2	1947.4	-0.95	**2,053**	-0.87	**2,229**	-0.08	**24,099**	0.04	**44,227**
3	1947.4	-1.65	**1,180**	-1.52	**1,281**	-0.14	**13,852**	0.08	**25,417**

*per 10 children (1 from each of the 10 grade 5 cohorts).

**Scenarios:** 1-The annual decline in obesity is maintained for the 2 intervention years then remains at the level reached in 2 years for another 8 years; 2- After the two intervention years, the effect starts waning by 5% per year; 3- The annual decline in obesity is maintained for 4 years then remains at the level reached in 4 years for another 6 years

**Fig 3:**
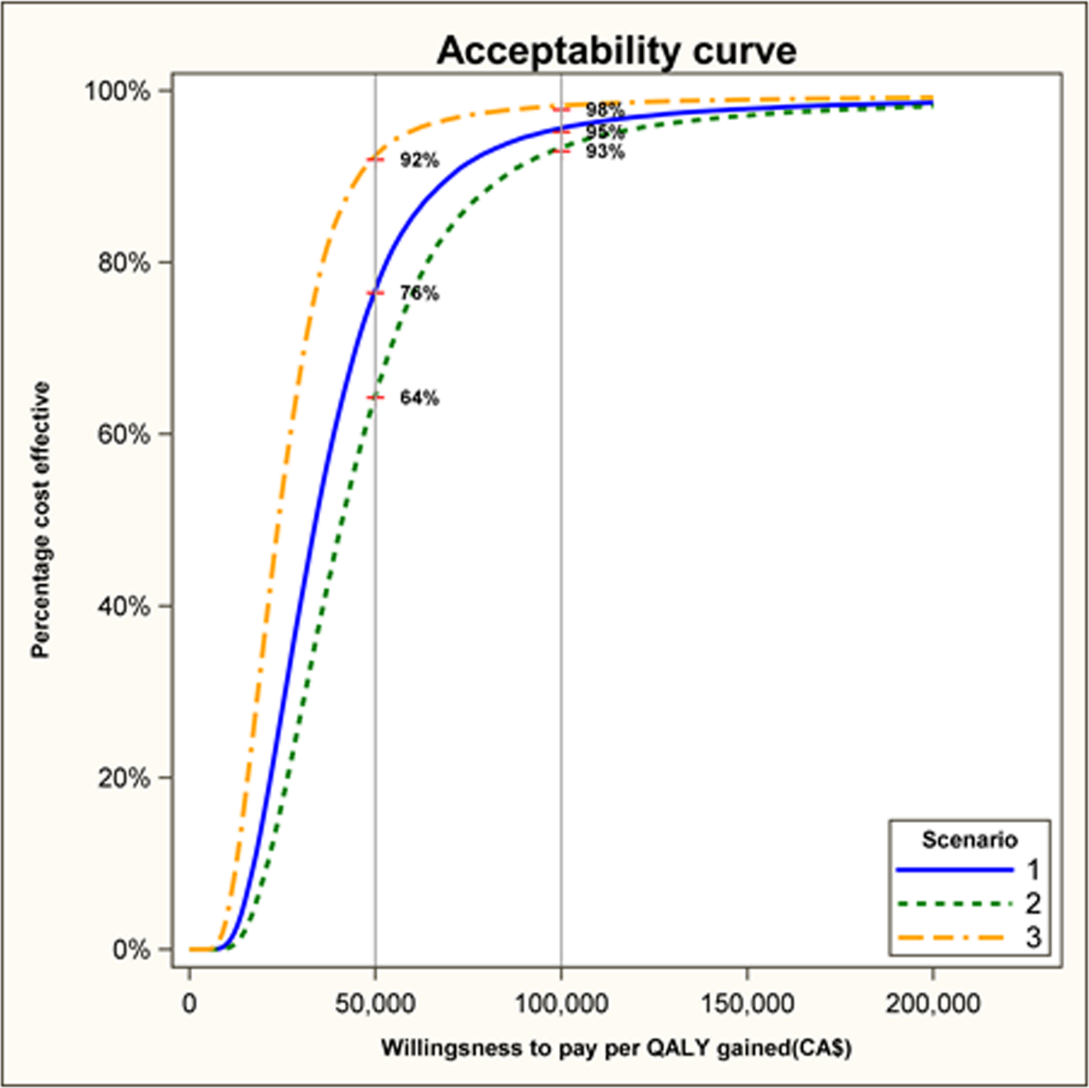
Probabilistic sensitivity analysis. Scenarios: 1-The annual decline in obesity is maintained for the 2 intervention years then remains at the level reached in 2 years for another 8 years 2- After the two intervention years, the effect starts waning by 5% per year 3- The annual decline in obesity is maintained for 4 years then remains at the level reached in 4 years for another 6 years.

Results of probabilistic sensitivity analysis (Fig 3), showed that this program has a high probability of being cost-effective based on the CA$50,000/QALY threshold, 76%, 64% and 92% in scenarios 1, 2, and 3, respectively. When using the CA$100,000 threshold, the corresponding estimates were, 95%, 93% and 98% of the time for scenario 1, 2 and 3, respectively (Fig 3).

## Discussion

We took a life course approach to evaluate the cost-effectiveness of APPLE Schools and showed that this program would generally be considered cost-effective at commonly cited threshold levels. In our base scenario, the ICER of APPLE Schools was estimated to be CA$1,555 per person year of excess weight prevented, CA$1,709 per person year of obesity prevented and CA$33,421 per QALY gained. Sensitivity analyses, showed that the APPLE schools program was cost effective (ICER ≤CA$100,000), 95% of the time per QALY gained in the first scenario and that the two alternative scenarios produced estimates that were not substantially different.

To the best of our knowledge, this is the first study to show cost effectiveness of a school-based health promotion in Canada. As countries and jurisdictions vary in their educational and health care systems, the present findings are particularly relevant to public health decision makers in Canada.

Our findings are consistent with previous economic evaluations of school-based programs for the prevention of obesity in other countries. In a recent systematic review, Korber estimated cost-effectiveness ratios for various school-based programs from a societal perspective, ranging from US$1,073 to US$669,1380 per QALY/DALY gained (in 2011 US dollars) [16]. The cost-effectiveness ratios were generally higher for evaluations based on longer time-horizons compared to those based on shorter time horizons. In programs evaluated on a lifetime, as we did, the cost effectiveness ratios ranged from US$15,478 to US$669,138 per QALY/DALY gained. At a threshhold of US$100,000 per QALY gained, three of these programs (Moodie et al 2013 = US$15,478; Rush et al 2014 = US$16,571 and Moodie et al 2010 = US$72,197) would be considered cost effective and two would not be cost-effective (Moodie et al 2011 = US$103,012 and Moodie et al 2009 = US$669,138) [16]. In comparison, our estimated cost-effective ratio for APPLE schools in 2011 US dollars, would be about US$28,302 per QALY gained, which is cost-effective at a threshold of US$100,000 per QALY gained.

In contrast, programs that were evaluated in a shorter time-horizon (10 to 25 years), had estimated cost effectiveness ratios (in 2011 US dollars) ranging from US$1,073 to US$6,179 per QALY gained (Brown et al 2007 = US$1,073; Wang et al 2011 = US$3,061 and Wang et al 2003 = US$6,179).

We showed APPLE Schools to be cost effective despite our evaluation being based on only its effect through changes in BMI without considering other positive outcomes of the program. For example, we miss the effects of reduction in salt on hypertension. We also did not consider cost savings associated with avoided health care services. Their consideration would have resulted in even stronger estimates for cost-effectiveness.

Furthermore, we also miss the health benefits that may arise from improvements in knowledge of health, attitudes, self-efficacy and leadership that are also targeted by the APPLE Schools [53–55]. Therefore, our estimates for the cost effectiveness ratio per QALY gained may be conservative. In addition, we also missed the benefit received by students who were in grade 6 when the APPLE schools started, because we only evaluated student cohorts at grade 5. Furthermore, our evaluation was based on only chronic diseases for which a published meta-analysis had shown significant relationships with weight status [27].

The study also has other limitations to be acknowledged. Whereas heights and weights of students attending APPLE Schools and general schools were measured, heights and weights from the surveys that were used to model the life course weight status transitions were self-reported. In addition, the quantification of effectiveness in this study was based on ecological change in the distribution of weight status of grade 5 students. We did not examine the effect of the program at individual level, because we did not follow the same students over time.

As with most analytical models, economic evaluation models are a simplification of reality with a number of assumptions and model inputs obtained from various sources[56]. Though we used probabilistic sensitivity analysis to address uncertainty in model input parameters, it cannot take into account all the possible sources of uncertainty [52].

In conclusion, based on our modeling approach, APPLE Schools appears to be a cost-effective intervention for obesity prevention and reduction of chronic disease risk over the lifetime of children attending APPLE Schools. Expanding the coverage and allocating resources towards school-based programs like the APPLE schools program, is likely to reduce the public health burden of obesity and chronic diseases.

## Supporting information

S1 Table Multinomial logistic regression model for Weight status transition probabilities. (DOCX)

S2 Table Effect of weight status on all-cause mortality. (DOCX)

S3 Table Effect of chronic diseases on mortality. (DOCX)

S4 Table Weight status distribution in Canada by age and sex. (DOCX)

S5 Table Prevalence of chronic diseases. (DOCX)

S6 Table Annual probability (incidence) of developing a given chronic disease in the general population. (DOCX)

S7 Table Effect of weight status on disease incidence. (DOCX)

S8 Table Impact of disease conditions and weight status on HRQOL. (DOCX)

S9 Table Total Expenditure in the 2 year intervention period 2008–2010. (DOCX)
