# Correction: Cost-effectiveness of a school-based health promotion program in Canada: A life-course modeling approach

**DOI:** 10.1371/journal.pone.0212084

**Published:** 2019-02-05

**Authors:** John Paul Ekwaru, Arto Ohinmaa, Bach Xuan Tran, Solmaz Setayeshgar, Jeffrey A. Johnson, Paul J. Veugelers

S1 Table is incomplete and contains errors. Please see the complete, correct S1 Table here.

## Supporting information

S1 Table Multinomial logistic regression model for Weight status transition probabilities. (DOCX)
