# Supplementary material for: Cost-effectiveness of a school-based health promotion program in Canada: A life-course modeling approach
Source: PLoS One. 2017 May 18;12(5):e0177848. doi: 10.1371/journal.pone.0177848 (PMC5436822; doi:10.1371/journal.pone.0177848)
Supplement: S1 Table — (DOCX) [file pone.0177848.s001.docx]

**S1 Table: Multinomial logistic regression model for Weight status transition probabilities.**

|  | **Outcome( weight status in 2 years)†** | | | |
| --- | --- | --- | --- | --- |
|  | ***Over weight*** | | ***Obese*** | |
| ***Variable*** | ***β*** | ***p-value*** | ***β*** | ***p-value*** |
| **Weight Status** |  |  |  |  |
| Underweight/Normal Weight | Ref. |  | Ref. |  |
| Over weight | 1.678 | <.0001 | 0.970 | 0.0020 |
| Obese | 0.473 | 0.0563 | 1.262 | 0.0002 |
| **Sex** |  |  |  |  |
| Male | Ref. |  | Ref. |  |
| Female | -.847 | <.0001 | -.460 | <.0001 |
| Age | 0.031 | <.0001 | -.103 | <.0001 |
| Age^2^ | -.000 | <.0001 | 0.001 | <.0001 |
| **Age*Weight Status** |  |  |  |  |
| Underweight/Normal Weight | Ref. |  | Ref. |  |
| Over weight | 0.077 | <.0001 | 0.200 | <.0001 |
| Obese | 0.164 | <.0001 | 0.372 | <.0001 |
| **Age^2^*Weight Status** |  |  |  |  |
| Underweight/Normal Weight | Ref. |  | Ref. |  |
| Over weight | -.001 | <.0001 | -.002 | <.0001 |
| Obese | -.002 | <.0001 | -.004 | <.0001 |
| **Age*Sex** |  |  |  |  |
| Male | Ref. |  | Ref. |  |
| Female | 0.008 | <.0001 | 0.006 | 0.0046 |

**†Normal weight is the reference outcome level.**
