# Supplementary material for: Cost-effectiveness of a school-based health promotion program in Canada: A life-course modeling approach
Source: PLoS One. 2017 May 18;12(5):e0177848. doi: 10.1371/journal.pone.0177848 (PMC5436822; doi:10.1371/journal.pone.0177848)
Supplement: S2 Table — (DOCX) [file pone.0177848.s002.docx]

**S2 Table: Effect of weight status on all-cause mortality**

| **Source** | **Weight Status** | **Relative Risk** | **95% CI** | |
| --- | --- | --- | --- | --- |
| Flegal et al., 2013 [[28](#_ENREF_28)] | Normal Weight – **reference** | 1.0 |  |  |
| *Meta-Analysis* | Over weight | 0.94 | 0.91 | 0.96 |
|  | Obesity | 1.18 | 1.12 | 1.25 |
