# Supplementary material for: Cost-effectiveness of a school-based health promotion program in Canada: A life-course modeling approach
Source: PLoS One. 2017 May 18;12(5):e0177848. doi: 10.1371/journal.pone.0177848 (PMC5436822; doi:10.1371/journal.pone.0177848)
Supplement: S3 Table — (DOCX) [file pone.0177848.s003.docx]

**S3 Table: Effect of chronic diseases on mortality**

| **Source** | **Disease** | **Extracted measure** | **Age Group** | **Male** | **Female** |
| --- | --- | --- | --- | --- | --- |
| Preis, et al., 2005[[29](#_ENREF_29)] | Diabetes | Mortality Ratio | 45-74 | 1.81 | 2.29 |
| Robitaille et al., 2012[[30](#_ENREF_30)] | Hypertension | Mortality Ratio | 20-24 | 4.2 | 4.2 |
|  |  |  | 25-29 | 4 | 4 |
|  |  |  | 30-34 | 2.7 | 2.7 |
|  |  |  | 35-39 | 2.4 | 2.4 |
|  |  |  | 40-44 | 2.4 | 2.4 |
|  |  |  | 45-49 | 1.9 | 1.9 |
|  |  |  | 50-54 | 1.8 | 1.8 |
|  |  |  | 55-59 | 1.6 | 1.6 |
|  |  |  | 60-64 | 1.5 | 1.5 |
|  |  |  | 65-69 | 1.5 | 1.5 |
|  |  |  | 70-74 | 1.4 | 1.4 |
|  |  |  | 75-79 | 1.2 | 1.2 |
|  |  |  | 80-84 | 1.2 | 1.2 |
|  |  |  | >=85 | 1.1 |  |
| Bronnum-Hansen, et al., 2001[[31](#_ENREF_31)] | Stroke | Standardized Mortality Ratio(SMR) | >=25 | 2.58 | 2.85 |
| **Used same values for stroke(CHD specific data not yet found)* | CHD | - | - | - | - |
| Canadian Cancer statistics 2015[[32](#_ENREF_32)] | Kidney cancer | Mortality Ratio | 15-99 | 1.49 | 1.45 |
|  | Pancreas cancer | Mortality Ratio | 15-99 | 12.5 | 12.5 |
|  | Colorectal cancer | Mortality Ratio | 15-99 | 1.56 | 1.54 |
|  | Breast cancer | Mortality Ratio | 15-99 | 1.25 | 1.14 |
|  | Body of Uterus cancer | Mortality Ratio | 15-99 |  | 1.18 |
|  | Ovary cancer | Mortality Ratio | 15-99 |  | 2.22 |
|  | Bladder cancer | Mortality Ratio | 15-99 | 1.35 | 1.4 |
| Vandentorren, et al., 2003[[33](#_ENREF_33)] | Asthma | Mortality Ratio | 25-59 | 1.15 | 1.22 |
| Nuesch, et al., 2011[[34](#_ENREF_34)] | Osteoarthritis | Mortality Ratio | >=35 | 1.58 | 1.52 |
