# Supplementary material for: Cost-effectiveness of a school-based health promotion program in Canada: A life-course modeling approach
Source: PLoS One. 2017 May 18;12(5):e0177848. doi: 10.1371/journal.pone.0177848 (PMC5436822; doi:10.1371/journal.pone.0177848)
Supplement: S4 Table — (DOCX) [file pone.0177848.s004.docx]

**S4 – Weight status distribution in Canada by age and sex.**

|  |  | ***Proportion*** | | |
| --- | --- | --- | --- | --- |
| ***Source*** | ***Age group*** | ***Normal Weight*** | ***Over Weight*** | ***Obese*** |
| Statistics Canada, 2010[[35](#_ENREF_35)] | Male |  |  |  |
|  | 6 – 11 | 0.758 | 0.172 | 0.071 |
|  | 12 – 17 | 0.707 | 0.169 | 0.124 |
|  | 18 – 39 | 0.442 | 0.362 | 0.196 |
|  | 40 – 59 | 0.206 | 0.519 | 0.275 |
|  | 60+ | 0.247 | 0.443 | 0.31 |
|  | Female |  |  |  |
|  | 6 – 11 | 0.779 | 0.163 | 0.058 |
|  | 12 – 17 | 0.738 | 0.18 | 0.083 |
|  | 18 – 39 | 0.524 | 0.229 | 0.247 |
|  | 40 – 59 | 0.446 | 0.31 | 0.244 |
|  | 60+ | 0.308 | 0.384 | 0.308 |
