# Supplementary material for: Cost-effectiveness of a school-based health promotion program in Canada: A life-course modeling approach
Source: PLoS One. 2017 May 18;12(5):e0177848. doi: 10.1371/journal.pone.0177848 (PMC5436822; doi:10.1371/journal.pone.0177848)
Supplement: S5 Table — (DOCX) [file pone.0177848.s005.docx]

**S5 Table: Prevalence of chronic diseases**

| *source* | *Chronic Disease* | *Age group* | *Male* | *Female* |
| --- | --- | --- | --- | --- |
| Statistics Canada , 2016 [[36](#_ENREF_36)] | Diabetes | <12 | 0.0060 | 0.0040 |
|  |  | 12-20 | 0.0060 | 0.0040 |
|  |  | 20-35 | 0.0110 | 0.0080 |
|  |  | 35-45 | 0.0230 | 0.0310 |
|  |  | 45-65 | 0.0660 | 0.1040 |
|  |  | 65+ | 0.1620 | 0.2060 |
|  | Hypertension | <12 | 0.0050 | 0.0050 |
|  |  | 12-20 | 0.0050 | 0.0050 |
|  |  | 20-35 | 0.0140 | 0.0310 |
|  |  | 35-45 | 0.0600 | 0.0870 |
|  |  | 45-65 | 0.2000 | 0.2680 |
|  |  | 65+ | 0.4880 | 0.4570 |
|  | Asthma | <12 | 0.0070 | 0.0070 |
|  |  | 12-20 | 0.0070 | 0.0070 |
|  |  | 20-35 | 0.0310 | 0.0290 |
|  |  | 35-45 | 0.0740 | 0.0570 |
|  |  | 45-65 | 0.2520 | 0.1690 |
|  |  | 65+ | 0.4850 | 0.3340 |
|  | Osteoarthritis | <12 | 0.0960 | 0.0830 |
|  |  | 12-20 | 0.0960 | 0.0830 |
|  |  | 20-35 | 0.1050 | 0.0820 |
|  |  | 35-45 | 0.0890 | 0.0550 |
|  |  | 45-65 | 0.0860 | 0.0640 |
|  |  | 65+ | 0.0870 | 0.0710 |
| Statistics Canada , CCHS 4.1 [[37](#_ENREF_37)] | Stroke | <12 | 0.0011 | 0.0008 |
|  |  | 12-20 | 0.0011 | 0.0008 |
|  |  | 20-35 | 0.0010 | 0.0008 |
|  |  | 35-45 | 0.0035 | 0.0026 |
|  |  | 45-65 | 0.0110 | 0.0098 |
|  |  | 65+ | 0.0527 | 0.0386 |
|  | CHD | <12 | 0.0069 | 0.0042 |
|  |  | 12-20 | 0.0069 | 0.0042 |
|  |  | 20-35 | 0.0059 | 0.0050 |
|  |  | 35-45 | 0.0145 | 0.0136 |
|  |  | 45-65 | 0.0602 | 0.0405 |
|  |  | 65+ | 0.2149 | 0.1633 |
| Ellisonet al, 2012 [[38](#_ENREF_38)] | Kidney Cancer | Overall | 0.0003 | 0.0002 |
|  | Pancreatic Cancer | Overall | 0.0001 | 0.0001 |
|  | Colorectal Cancer | Overall | 0.0010 | 0.0008 |
|  | Breast Cancer | Overall | 0.0000 | 0.0023 |
|  | Endometrial Cancer | Overall | 0.0000 | 0.0001 |
|  | Ovarian Cancer | Overall | 0.0000 | 0.0002 |
|  | Gallbladder Cancer | Overall | 0.0004 | 0.0001 |
