# Supplementary material for: Cost-effectiveness of a school-based health promotion program in Canada: A life-course modeling approach
Source: PLoS One. 2017 May 18;12(5):e0177848. doi: 10.1371/journal.pone.0177848 (PMC5436822; doi:10.1371/journal.pone.0177848)
Supplement: S6 Table — (DOCX) [file pone.0177848.s006.docx]

**S6 Table: Annual probability (incidence) of developing a given chronic disease in the general population.**

|  |  | | **Probability** | |
| --- | --- | --- | --- | --- |
| **Source** | **Chronic disease** | **Age group** | **Females** | **Males** |
| Pelletier et al, 2012 [[39](#_ENREF_39)] | Diabetes | 1-19 | 0.000400 | 0.000400 |
|  |  | 20-24 | 0.000600 | 0.000900 |
|  |  | 25-29 | 0.001099 | 0.001499 |
|  |  | 30-34 | 0.002198 | 0.002397 |
|  |  | 35-39 | 0.003793 | 0.003295 |
|  |  | 40-44 | 0.005883 | 0.004291 |
|  |  | 45-49 | 0.008166 | 0.005783 |
|  |  | 50-54 | 0.011533 | 0.008266 |
|  |  | 55-59 | 0.015380 | 0.011138 |
|  |  | 60-64 | 0.019115 | 0.014001 |
|  |  | 65-69 | 0.022347 | 0.016561 |
|  |  | 70-74 | 0.023128 | 0.017643 |
|  |  | 75-79 | 0.022444 | 0.017741 |
|  |  | 80-84 | 0.020683 | 0.017053 |
|  |  | 85+ | 0.016069 | 0.013508 |
|  |  |  |  |  |
| Robitaille et al, 2012 [[30](#_ENREF_30)] | Hypertension | 0-19 | 0.000000 | 0.000000 |
|  |  | 20–24 | 0.001998 | 0.001998 |
|  |  | 25–29 | 0.002996 | 0.002996 |
|  |  | 30–34 | 0.003992 | 0.005982 |
|  |  | 35–39 | 0.006976 | 0.008960 |
|  |  | 40–44 | 0.011928 | 0.013902 |
|  |  | 45–49 | 0.018821 | 0.020781 |
|  |  | 50–54 | 0.026639 | 0.029554 |
|  |  | 55–59 | 0.033428 | 0.039211 |
|  |  | 60–64 | 0.044958 | 0.050671 |
|  |  | 65–69 | 0.059177 | 0.061995 |
|  |  | 70–74 | 0.072257 | 0.071328 |
|  |  | 75–79 | 0.082406 | 0.078728 |
|  |  | 80–84 | 0.087895 | 0.080569 |
|  |  | 85+ | 0.073184 | 0.066673 |
|  |  |  |  |  |
| Johansen et al, 2006 [[40](#_ENREF_40)] | Stroke | <20 | 0.000110 | 0.000100 |
|  |  | 20-44 | 0.000110 | 0.000100 |
|  |  | 45-64 | 0.000710 | 0.001019 |
|  |  | 65-79 | 0.004072 | 0.005515 |
|  |  | 80+ | 0.012630 | 0.014011 |
|  |  |  |  |  |
| Mozaffarian et al, 2015 [[41](#_ENREF_41)] | CHD | <35 | 0.009950 | 0.024690 |
|  |  | 35-44 | 0.009950 | 0.024690 |
|  |  | 45-54 | 0.034395 | 0.072257 |
|  |  | 55-64 | 0.058235 | 0.117503 |
|  |  | 65-74 | 0.067606 | 0.117503 |
|  |  | 75-84 | 0.095163 | 0.113080 |
|  |  | 85+ | 0.104166 | 0.076884 |
| Gershon et al, 2010 [[43](#_ENREF_43)] | Asthma | 0-4 | 0.035553 | 0.025957 |
|  |  | 5-9 | 0.012027 | 0.009554 |
|  |  | 10-14 | 0.005385 | 0.005883 |
|  |  | 15-39 | 0.002198 | 0.003594 |
|  |  | 40-69 | 0.002198 | 0.003295 |
|  |  | 70+ | 0.003095 | 0.003394 |
| Kopec et al, 2007 [[44](#_ENREF_44)] | Osteoarthritis | 0–19 | 0.000000 | 0.000000 |
|  |  | 20–24 | 0.002198 | 0.002198 |
|  |  | 25–29 | 0.003295 | 0.002996 |
|  |  | 30–34 | 0.004589 | 0.004589 |
|  |  | 35–39 | 0.007373 | 0.006777 |
|  |  | 40–44 | 0.010841 | 0.009455 |
|  |  | 45–49 | 0.017053 | 0.012521 |
|  |  | 50–54 | 0.024983 | 0.018035 |
|  |  | 55–59 | 0.033815 | 0.022444 |
|  |  | 60–64 | 0.040267 | 0.028098 |
|  |  | 65–69 | 0.045531 | 0.032655 |
|  |  | 70–74 | 0.050576 | 0.038249 |
|  |  | 75–79 | 0.055973 | 0.042855 |
|  |  | 80–84 | 0.058989 | 0.050576 |
|  |  | 85–90 | 0.056822 | 0.051999 |
|  |  | 90+ | 0.061338 | 0.056727 |
|  |  |  |  |  |
| Statistics Canada [[42](#_ENREF_42)] | Kidney Cancer | 0-4 | 0.000022 | 0.000015 |
|  |  | 5-9 | 0.000000 | 0.000005 |
|  |  | 10-14 | 0.000000 | 0.000000 |
|  |  | 15-19 | 0.000000 | 0.000004 |
|  |  | 20-24 | 0.000004 | 0.000004 |
|  |  | 25-29 | 0.000013 | 0.000008 |
|  |  | 30-34 | 0.000013 | 0.000026 |
|  |  | 35-39 | 0.000039 | 0.000040 |
|  |  | 40-44 | 0.000042 | 0.000113 |
|  |  | 45-49 | 0.000094 | 0.000163 |
|  |  | 50-54 | 0.000141 | 0.000250 |
|  |  | 55-59 | 0.000208 | 0.000335 |
|  |  | 60-64 | 0.000279 | 0.000488 |
|  |  | 65-69 | 0.000278 | 0.000640 |
|  |  | 70-74 | 0.000371 | 0.000780 |
|  |  | 75-79 | 0.000384 | 0.000787 |
|  |  | 80-84 | 0.000486 | 0.000813 |
|  |  | 85+ | 0.000451 | 0.000701 |
|  | Pancreatic Cancer | 0-4 | 0.000000 | 0.000000 |
|  |  | 5-9 | 0.000000 | 0.000000 |
|  |  | 10-14 | 0.000000 | 0.000000 |
|  |  | 15-19 | 0.000000 | 0.000000 |
|  |  | 20-24 | 0.000004 | 0.000000 |
|  |  | 25-29 | 0.000000 | 0.000004 |
|  |  | 30-34 | 0.000000 | 0.000009 |
|  |  | 35-39 | 0.000009 | 0.000018 |
|  |  | 40-44 | 0.000021 | 0.000033 |
|  |  | 45-49 | 0.000045 | 0.000055 |
|  |  | 50-54 | 0.000085 | 0.000103 |
|  |  | 55-59 | 0.000141 | 0.000180 |
|  |  | 60-64 | 0.000221 | 0.000281 |
|  |  | 65-69 | 0.000339 | 0.000442 |
|  |  | 70-74 | 0.000524 | 0.000580 |
|  |  | 75-79 | 0.000591 | 0.000679 |
|  |  | 80-84 | 0.000741 | 0.000830 |
|  |  | 85+ | 0.000733 | 0.000935 |
|  | Colorectal Cancer | 0-4 | 0.000000 | 0.000000 |
|  |  | 5-9 | 0.000006 | 0.000000 |
|  |  | 10-14 | 0.000005 | 0.000000 |
|  |  | 15-19 | 0.000014 | 0.000004 |
|  |  | 20-24 | 0.000013 | 0.000017 |
|  |  | 25-29 | 0.000025 | 0.000033 |
|  |  | 30-34 | 0.000059 | 0.000060 |
|  |  | 35-39 | 0.000088 | 0.000084 |
|  |  | 40-44 | 0.000163 | 0.000167 |
|  |  | 45-49 | 0.000269 | 0.000303 |
|  |  | 50-54 | 0.000503 | 0.000566 |
|  |  | 55-59 | 0.000664 | 0.000987 |
|  |  | 60-64 | 0.001048 | 0.001509 |
|  |  | 65-69 | 0.001482 | 0.002403 |
|  |  | 70-74 | 0.001917 | 0.003159 |
|  |  | 75-79 | 0.002518 | 0.003783 |
|  |  | 80-84 | 0.003320 | 0.004480 |
|  |  | 85+ | 0.003312 | 0.004526 |
|  | Breast Cancer | 0-4 | 0.000000 | 0.000000 |
|  |  | 5-9 | 0.000000 | 0.000000 |
|  |  | 10-14 | 0.000000 | 0.000000 |
|  |  | 15-19 | 0.000005 | 0.000000 |
|  |  | 20-24 | 0.000017 | 0.000000 |
|  |  | 25-29 | 0.000079 | 0.000004 |
|  |  | 30-34 | 0.000224 | 0.000000 |
|  |  | 35-39 | 0.000557 | 0.000000 |
|  |  | 40-44 | 0.001040 | 0.000004 |
|  |  | 45-49 | 0.001630 | 0.000000 |
|  |  | 50-54 | 0.002043 | 0.000007 |
|  |  | 55-59 | 0.002334 | 0.000017 |
|  |  | 60-64 | 0.003023 | 0.000025 |
|  |  | 65-69 | 0.003535 | 0.000038 |
|  |  | 70-74 | 0.003742 | 0.000054 |
|  |  | 75-79 | 0.003569 | 0.000072 |
|  |  | 80-84 | 0.003320 | 0.000085 |
|  |  | 85+ | 0.003075 | 0.000094 |
|  | Endometrial Cancer | 0-4 | 0.000000 | 0.000000 |
|  |  | 5-9 | 0.000000 | 0.000000 |
|  |  | 10-14 | 0.000000 | 0.000000 |
|  |  | 15-19 | 0.000000 | 0.000000 |
|  |  | 20-24 | 0.000000 | 0.000000 |
|  |  | 25-29 | 0.000000 | 0.000000 |
|  |  | 30-34 | 0.000000 | 0.000000 |
|  |  | 35-39 | 0.000004 | 0.000000 |
|  |  | 40-44 | 0.000008 | 0.000000 |
|  |  | 45-49 | 0.000011 | 0.000000 |
|  |  | 50-54 | 0.000011 | 0.000000 |
|  |  | 55-59 | 0.000017 | 0.000000 |
|  |  | 60-64 | 0.000014 | 0.000000 |
|  |  | 65-69 | 0.000018 | 0.000000 |
|  |  | 70-74 | 0.000032 | 0.000000 |
|  |  | 75-79 | 0.000020 | 0.000000 |
|  |  | 80-84 | 0.000024 | 0.000000 |
|  |  | 85+ | 0.000068 | 0.000000 |
|  | Ovarian Cancer | 0-4 | 0.000000 | 0.000000 |
|  |  | 5-9 | 0.000000 | 0.000000 |
|  |  | 10-14 | 0.000005 | 0.000000 |
|  |  | 15-19 | 0.000009 | 0.000000 |
|  |  | 20-24 | 0.000021 | 0.000000 |
|  |  | 25-29 | 0.000025 | 0.000000 |
|  |  | 30-34 | 0.000025 | 0.000000 |
|  |  | 35-39 | 0.000048 | 0.000000 |
|  |  | 40-44 | 0.000092 | 0.000000 |
|  |  | 45-49 | 0.000165 | 0.000000 |
|  |  | 50-54 | 0.000214 | 0.000000 |
|  |  | 55-59 | 0.000224 | 0.000000 |
|  |  | 60-64 | 0.000289 | 0.000000 |
|  |  | 65-69 | 0.000357 | 0.000000 |
|  |  | 70-74 | 0.000443 | 0.000000 |
|  |  | 75-79 | 0.000453 | 0.000000 |
|  |  | 80-84 | 0.000473 | 0.000000 |
|  |  | 85+ | 0.000429 | 0.000000 |
|  | Gallbladder Cancer | 0-4 | 0.000000 | 0.000000 |
|  |  | 5-9 | 0.000000 | 0.000000 |
|  |  | 10-14 | 0.000000 | 0.000000 |
|  |  | 15-19 | 0.000000 | 0.000000 |
|  |  | 20-24 | 0.000000 | 0.000000 |
|  |  | 25-29 | 0.000004 | 0.000000 |
|  |  | 30-34 | 0.000004 | 0.000000 |
|  |  | 35-39 | 0.000000 | 0.000000 |
|  |  | 40-44 | 0.000000 | 0.000000 |
|  |  | 45-49 | 0.000007 | 0.000004 |
|  |  | 50-54 | 0.000007 | 0.000007 |
|  |  | 55-59 | 0.000025 | 0.000013 |
|  |  | 60-64 | 0.000034 | 0.000020 |
|  |  | 65-69 | 0.000054 | 0.000045 |
|  |  | 70-74 | 0.000065 | 0.000045 |
|  |  | 75-79 | 0.000089 | 0.000083 |
|  |  | 80-84 | 0.000121 | 0.000119 |
|  |  | 85+ | 0.000090 | 0.000094 |
