# Supplementary material for: Cost-effectiveness of a school-based health promotion program in Canada: A life-course modeling approach
Source: PLoS One. 2017 May 18;12(5):e0177848. doi: 10.1371/journal.pone.0177848 (PMC5436822; doi:10.1371/journal.pone.0177848)
Supplement: S7 Table — (DOCX) [file pone.0177848.s007.docx]

**S7 Table: Effect of weight status on disease incidence**

| **Source** | **Chronic disease** | **Incident Rate Ratio(95% CI)** | |
| --- | --- | --- | --- |
| **Guh et al., 2009 [**[**25**](#_ENREF_25)**]**  ***Meta-Analysis*** |  | **Overweight** | **Obese** |
|  | **Cancers** |  |  |
|  | Breast - *Female* | 1.08(1.03 - 1.14) | 1.13(1.05 - 1.22) |
|  | Endometrial - *Female* | 1.53(1.45 - 1.61) | 3.22(2.91 - 3.56) |
|  | Ovarian - *Female* | 1.18(1.12 - 1.23) | 1.28(1.20 - 1.36) |
|  | Colorectal |  |  |
|  | *Female* | 1.45(1.30 - 1.62) | 1.66(1.52 - 1.81) |
|  | *Male* | 1.51(1.37 - 1.67) | 1.95(1.59 - 2.39) |
|  | Kidney |  |  |
|  | *Female* | 1.82(1.68 - 1.98) | 2.64(2.39 - 2.90) |
|  | *Male* | 1.40(1.31 - 1.49) | 1.82(1.61 - 2.05) |
|  | Pancreatic |  |  |
|  | *Female* | 1.24(0.98 - 1.56) | 1.60(1.17 - 2.20) |
|  | *Male* | 1.28(0.94 - 1.75) | 2.29(1.65 - 3.19) |
|  | ***Gallbladder Disease*** |  |  |
|  | *Female* | 1.44(1.05 - 1.98) | 2.32(1.17 - 4.57) |
|  | *Male* | 1.09(0.87 - 1.37) | 1.43(1.04 - 1.96) |
|  | **Type II Diabetes** |  |  |
|  | Female | 3.92(3.10 - 4.97) | 12.41(9.03 - 17.06) |
|  | Male | 2.40(2.12 - 2.72) | 6.74(5.55 - 8.19) |
|  | **Hypertension** |  |  |
|  | *Female* | 1.65(1.24 - 2.19) | 2.42(1.59 - 3.67) |
|  | *Male* | 1.28(1.10 - 1.50) | 1.84(1.51 - 2.24) |
|  | **Stroke** |  |  |
|  | *Female* | 1.15(1.00 - 1.32) | 1.49(1.27 - 1.74) |
|  | *Male* | 1.23(1.13 - 1.34) | 1.51(1.33 - 1.72) |
|  | **Coronary Artery Disease (CHD)** |  |  |
|  | *Female* | 1.80(1.64 - 1.98) | 3.10(2.81 - 3.43) |
|  | *Male* | 1.29(1.18 - 1.41) | 1.72(1.51 - 1.96) |
|  | **Asthma** |  |  |
|  | *Female* | 1.25(1.05 - 1.49) | 1.78(1.36 - 2.32) |
|  | *Male* | 1.20(1.08 - 1.33) | 1.43(1.14 - 1.79) |
|  | **Osteoarthritis** |  |  |
|  | *Female* | 1.80(1.75 - 1.85) | 1.96(1.88 - 2.04) |
|  | *Male* | 2.76(2.05 - 3.70) | 4.20(2.76 - 6.41) |
