# Supplementary material for: Cost-effectiveness of a school-based health promotion program in Canada: A life-course modeling approach
Source: PLoS One. 2017 May 18;12(5):e0177848. doi: 10.1371/journal.pone.0177848 (PMC5436822; doi:10.1371/journal.pone.0177848)
Supplement: S8 Table — (DOCX) [file pone.0177848.s008.docx]

**S8 Table: Impact of disease conditions and weight status on HRQOL**

| ***Source*** | ***Disease/Weight Status*** | ***Impact of disease condition*** | ***95% CI*** |
| --- | --- | --- | --- |
| Schultz et al., 2003 [[47](#_ENREF_47)] | Diabetes | -0.06 | -0.07, -0.04 |
|  | Hypertension | -0.01 | -0.02, 0.00 |
|  | Stroke | -0.17 | -0.22, -0.13 |
|  | CHD | -0.06 | -0.08, -0.05 |
|  | Cancers | -0.02 | -0.04, 0.00 |
|  | Asthma | -0.02 | -0.03, -0.01 |
|  | Osteoarthritis | -0.09 | -0.10, -0.07 |
|  |  |  |  |
| Jia et al., 2005[[48](#_ENREF_48)] | Over weight | -0.013 |  |
|  | *Class I Obesity* | *-0.033* |  |
|  | *Class II Obesity* | *-0.073* |  |
|  | Obesity(Weighted average) | -0.047 |  |
