# Supplementary material for: Cost-effectiveness of a school-based health promotion program in Canada: A life-course modeling approach
Source: PLoS One. 2017 May 18;12(5):e0177848. doi: 10.1371/journal.pone.0177848 (PMC5436822; doi:10.1371/journal.pone.0177848)
Supplement: S9 Table — (DOCX) [file pone.0177848.s009.docx]

**S9 Table: Total Expenditure in the 2 year intervention period 2008-2010**

| **Item** | **Amount (2008 CA$)** |
| --- | --- |
| Admin Staff Costs | 164,185 |
| Intervention staff Costs | 1,796,311 |
| Miscellaneous | 296,916 |
| Total | 2,257,412 |
| *Total Number of students* | *7,963* |
| *Cost per student* | *284* |
