# Supplementary material for: Correction: Cost-effectiveness of a school-based health promotion program in Canada: A life-course modeling approach
Source: PLoS One. 2019 Feb 5;14(2):e0212084. doi: 10.1371/journal.pone.0212084 (PMC6363220; doi:10.1371/journal.pone.0212084)
Supplement: S1 Table — (DOCX) [file pone.0212084.s001.docx]

**S1 Table: Multinomial logistic regression model for Weight status transition probabilities.**

|  | **Outcome( weight status in 2 years)†** | | | |
| --- | --- | --- | --- | --- |
|  | *Over weight* | | *Obese* | |
| *Variable* | *β* | *p-value* | *β* | *p-value* |
| Intercept | -2.1049 | <.0001 | -2.9330 | <.0001 |
| **Weight Status** |  |  |  |  |
| Underweight/Normal Weight | Ref. |  | Ref. |  |
| Over weight | 1.6776 | <.0001 | 0.9695 | 0.0020 |
| Obese | 0.4725 | 0.0563 | 1.2619 | 0.0002 |
| **Sex** |  |  |  |  |
| Male | Ref. |  | Ref. |  |
| Female | -0.8467 | <.0001 | -0.4598 | <.0001 |
| Age | 0.0312 | <.0001 | -0.1030 | <.0001 |
| Age^2^ | -0.0004 | <.0001 | 0.0010 | <.0001 |
| **Age*Weight Status** |  |  |  |  |
| Underweight/Normal Weight | Ref. |  | Ref. |  |
| Over weight | 0.0767 | <.0001 | 0.2003 | <.0001 |
| Obese | 0.1642 | <.0001 | 0.3715 | <.0001 |
| **Age^2^*Weight Status** |  |  |  |  |
| Underweight/Normal Weight | Ref. |  | Ref. |  |
| Over weight | -0.0007 | <.0001 | -0.0021 | <.0001 |
| Obese | -0.0015 | <.0001 | -0.0036 | <.0001 |
| **Age*Sex** |  |  |  |  |
| Male | Ref. |  | Ref. |  |
| Female | 0.0078 | <.0001 | 0.0063 | 0.0046 |

**†Normal weight is the reference outcome level.**
